# The Role of Mitochondrial ROS in Neoplastic Transformations, Progression and Therapeutic Targeting

**DOI:** 10.32604/or.2026.083159

**Published:** 2026-08-13

**Authors:** Bharath Kumar Velmurugan, Shu Hui Lin, Chih-Yang Huang, Ming-Ju Hsieh, Rathinasamy Baskaran

**Affiliations:** 1 Maitocon Laboratory, Tirupur, Tamil Nadu, India; 2 Department of Surgical Pathology, Changhua Christian Hospital, Changhua, Taiwan; 3 Department of Medical Laboratory Science and Biotechnology, Central Taiwan University of Science and Technology, Taichung, Taiwan; 4 Department of Post-Baccalaureate Medicine, College of Medicine, National Chung Hsing University, Taichung, Taiwan; 5 Department of Precision Chinese Herbal Medicine and Molecular Medicine Research, E-Da Cancer Hospital, E-Da Healthcare Group, Kaohsiung, Taiwan; 6 School of Chinese Medicine for Post Baccalaureate, I-Shou University, Kaohsiung, Taiwan; 7 Department of Medical Research, China Medical University Hospital, China Medical University, Taichung, Taiwan; 8 Department of Medical Laboratory Science and Biotechnology, Asia University, Taichung, Taiwan; 9 Graduate Institute of Biomedical Sciences, China Medical University, Taichung, Taiwan; 10 Cardiovascular and Mitochondrial Related Disease Research Center, Hualien Tzu Chi Hospital, Buddhist Tzu Chi Medical Foundation, Hualien, Taiwan; 11 Oral Cancer Research Center, Changhua Christian Hospital, Changhua, Taiwan; 12 Graduate Institute of Clinical Medicine, College of Medicine, National Chung Hsing University, Taichung, Taiwan

**Keywords:** Cancer, reactive oxygen species, mitochondria, oncometabolites, electron transport chain

## Abstract

Mitochondria are central regulators of cellular metabolism and survival and play a pivotal role in cancer development and progression through the production of reactive oxygen species (ROS), control of calcium homeostasis, regulation of autophagy, and modulation of cell death pathways. Mitochondria-derived ROS (mtROS) act as signaling mediators that influence tumor initiation, proliferation, metabolic reprogramming, metastasis, and therapeutic resistance by altering redox homeostasis, damaging mitochondrial DNA, and reshaping the tumor microenvironment. In addition to meeting the bioenergetic and biosynthetic requirements of rapidly proliferating cancer cells, mitochondrial metabolism modulates immune responses and supports cancer cell adaptation to hypoxia and nutrient deprivation. Accumulating evidence also highlights the dual role of mtROS, which can promote tumor progression at moderate levels yet trigger oxidative stress-induced cell death when excessively increased, making mitochondrial redox signaling an attractive therapeutic target. This review summarizes the major sources and regulation of mtROS, their involvement in cancer-associated signaling pathways, mitochondrial calcium dynamics, metabolic adaptations, and resistance to anticancer therapies, and discusses current and emerging mitochondrial-targeted strategies aimed at exploiting mtROS signaling to improve cancer treatment outcomes.

## 1 Introduction

In cancer, extensive alterations at the genomic, epigenomic, transcriptomic, and proteomic levels converge on a reprogrammed metabolism that supports malignant growth and survival in a microenvironment characterized by hypoxia, nutrient limitation, and immune surveillance. PET (positron emission tomography) and metabolomic studies show that tumor cells engage multiple, interconnected metabolic programs rather than a single dominant pathway, enabling sustained biomass production, redox balance, and energy supply [1]. Within this context, mitochondria are now recognized as central hubs that not only generate ATP and biosynthetic precursors but also produce ROS, modulate calcium signaling, and coordinate cell death and autophagy, making mtROS critical integrators of cancer cell fate [2].

Over the past decade, excellent reviews have synthesized the pleiotropic functions of ROS in cancer, the hallmarks of cancer metabolism, and the opportunities and limitations of targeting mitochondrial metabolism [2,3,4]. However, these works often consider ROS in a global manner or treat mitochondrial metabolism and ROS-based therapies without explicitly integrating mtROS, mitochondrial DNA alterations, and recent clinical experience with oxidative phosphorylation (OXPHOS) targeted agents [5]. In this review, we therefore focus on mtROS as integrators of cancer metabolism, signaling, and stress responses across three interconnected levels: neoplastic transformation, tumor progression, and therapeutic targeting. We first summarize sources and regulation of mtROS in cancer, then discuss their roles in mitochondrial DNA instability, oncogenic signaling, metabolic reprogramming, calcium homeostasis, and tumor progression, and finally evaluate therapeutic strategies that exploit mitochondrial redox vulnerabilities.

## Definitions, ROS Species, and Scope of mtROS

In this review, we use the term ROS to refer primarily to oxygen-derived oxidants generated within mitochondria, unless explicitly stated otherwise. The major mtROS species considered are superoxide (O_2_^−^), which is produced when single electrons leak from redox centers in the tricarboxylic acid (TCA) cycle or electron transport chain (ETC), and hydrogen peroxide (H_2_O_2_), which arises from superoxide dismutation by superoxide dismutase (SOD) and can diffuse across membranes to reach cytosolic and nuclear targets [6,7]. Highly reactive downstream species such as hydroxyl radical (•OH) and peroxynitrite (ONOO^—^) are also generated but are generally short-lived and act close to their site of formation. We distinguish mtROS, which originate from mitochondrial enzymes and the ETC and are initially confined to the mitochondrial matrix or inner membrane space, from non-mtROS, such as those produced by NADPH oxidases (NOX) at the plasma membrane or in endomembranes [8]. While NOX-derived ROS clearly contribute to tumor biology, our primary focus is on mtROS; instances in which NOX or other non-mitochondrial sources are implicated are explicitly indicated. Throughout the manuscript, we also emphasize that the functional consequences of ROS depend on species, concentration, subcellular compartmentalization matrix versus intermembrane space (IMS) versus cytosolic/nuclear and local antioxidant capacity.

Mitochondria generate ROS predominantly through electron leakage at redox centers in the TCA cycle and ETC, but the steady-state mtROS output in cancer cells is determined not only by these biochemical sources but also by compartmentalized antioxidant systems and extrinsic cues such as hypoxia, oncogene activation, and mitochondrial calcium flux [6,7]. This section summarizes canonical mtROS-producing sites and antioxidant circuits, and highlights how cancer-specific alterations in metabolism, mtDNA, and the tumor microenvironment shift mitochondrial redox homeostasis in ways that can promote either neoplastic transformation or tumor progression depending on context.

## 2 Sources and Regulation of Mitochondrial ROS in Cancer

### 2.1 The Tricarboxylic Acid Cycle (TCA Cycle)

The TCA cycle contains multiple flavoproteins and iron–sulfur cluster enzymes that can leak electrons to oxygen and generate superoxide under conditions of high flux or impaired downstream electron transfer. Enzymes such as pyruvate dehydrogenase, α-ketoglutarate dehydrogenase, and acyl-CoA dehydrogenases are recognized as potential mtROS sources, particularly when the ETC is saturated or partially inhibited, leading to accumulation of reduced redox centers and increased electron leak [9].

### 2.2 Electron Transport Chain (ETC) Cycle

Electrons derived from the TCA cycle enter the ETC, where complexes I and III are the major sites of mtROS production. Under physiological conditions, only a small fraction of oxygen consumed by mitochondria is converted to superoxide at these complexes, but factors such as elevated membrane potential, substrate overload, or partial complex inhibition can markedly increase electron leak and ROS generation. Ubiquinone and its semiquinone intermediate play a central role in this process, as delays in electron transfer at complex III increase the probability of superoxide formation [10].

### 2.3 The Subcellular Antioxidant System

Subcellular antioxidant systems tightly regulate mtROS levels and compartment-specific redox signaling. Within mitochondria, superoxide dismutase converts superoxide to hydrogen peroxide, which is further detoxified by glutathione peroxidases, peroxiredoxins, and the thioredoxin system using reducing equivalents from NADPH. Cancer cells frequently upregulate glutathione (GSH), thioredoxin (TRX), and Nuclear Factor Erythroid 2-Related Factor 2 (NRF2) dependent antioxidant programs, which help buffer chronic mtROS elevation but also contribute to therapy resistance and shape metastatic potential [11,12,13].

### 2.4 Hypoxia and ROS

A glycolytic shift is exhibited by most tumors, but mitochondrial oxygen consumption still remains substantial [14]. This consumption of oxygen shows a metabolic shift towards utilizing glucose for redox and biosynthetic processes. Generally, as tumors develop hypoxia, their growth speeds up compared to the vascular supply. In the mitochondrial matrix, during hypoxia, non-specific ROS generation declines and increases when hyperoxic [15,16]. But the release of ROS during hypoxia from complex III to the IMS increases significantly, which has been established by studies on oxidant signaling [17,18,19,20,21], deletion or suppression studies, and antioxidant studies dealing with *in-vivo* tumor growth [22,23], even though the exact mechanism is still far away. However, a possible insight gained from these studies is the outer binding site of ubiquinone in complex III, wherein low oxygen concentrations may lengthen the lifetime of the semiquinone radical, which may increase the generation of superoxides by facilitating optimal conditions for its generation in an oxygen-low environment [24]. The oxidant stress induced by hypoxia is broken down by the mitochondrial IMS when targeted by a hydrogen peroxide scavenger in the cytosol and compartment targeted to repel the H1F-1α stabilization of the cytosol [25]. In a hypoxic environment, the extensive generation of mtROS points towards a crucial aspect for tumor growth, reprogramming shifts in metabolism and cell survival.

## 3 Mitochondrial ROS and Mitochondrial DNA in Neoplastic Transformation

### 3.1 Combined Effect of mtROS and Mitochondrial DNA

Early neoplastic transformation is tightly linked to mtROS-driven damage and signaling events that accumulate before clinically apparent tumor progression. mtROS-induced mtDNA mutations altered mtDNA copy number, and changes in mitochondrial-nuclear communication can remodel respiratory chain function and redox state, thereby favoring oncogenic pathways such as Phosphatidylinositol 3-kinase (PI3K), Protein kinase B (AKT), and Mitogen-Activated Protein Kinase (MAPK) while promoting an oxidized cytosolic thiol environment and genomic instability [23,26]. In this section, we discuss how mtROS interact with mtDNA to initiate or accelerate tumorigenesis, distinguishing these early transformation events from the later ROS-dependent processes that sustain tumor growth and metastasis.

mtDNA remains an important target involved in mtROS for the initiation of cancer, as mutations on them propagates tumorogenesis [27]. mtDNA is maternally inherited, so mutations occurring on them can inflict the germline or inherited cancer types. They lack the protective barrier of histones as found in nuclear DNA, for which they have limited proofreading capacity and are profoundly prone to ROS attack [28]. Also, the mtDNA mutation rates are more frequent as compared to nuclear DNA, comprising deletions, point mutations, insertions, and deletions changing the mtDNA copy number. Heteroplasmy results from when mutations are passed on to the daughter cells along with the existing mtDNA. Sometimes, a subset of tumor cells drifts away during cell division (on dichotomous division of wild-type and mutant mitochondria) towards a state of homoplasmy. A substantial amount of homoplasmic and heteroplasmic mutations of mtDNA in different tissues of the same individual are found in both normal and cancer cells.

Large-scale sequencing studies have revealed that mtDNA mutations are common not only in tumors but also in normal tissues, with substantial inter-individual and tissue-specific variability in mtDNA copy number, heteroplasmy levels, and mutational spectra [4,26]. In many cancers, the mtDNA landscape includes a mixture of clearly pathogenic variants in respiratory complex genes and numerous synonymous or non-coding changes whose functional impact is uncertain, making it challenging to distinguish bona fide driver mutations from passengers that hitchhike during clonal expansion. The phenotypic consequences of a given mtDNA mutation are further modulated by its heteroplasmy level, nuclear genetic background, and tissue of origin, so that variants that are neutral in one context may be deleterious or tumor-promoting in another.

### 3.2 ROS Generation by mtDNA Mutations

Existing evidence indicates that a subset of mtDNA mutations is enriched in tumors and can modulate tumorigenic phenotypes in experimental systems, for example, when specific pathogenic mtDNA variants are introduced into or removed from cybrid models [23,29]. These studies demonstrate that certain mtDNA mutations are sufficient to alter respiratory chain function, mtROS production, and susceptibility to apoptosis, thereby influencing growth behavior *in vitro* and *in vivo*. However, only a small fraction of the mtDNA variants observed in human cancers has been functionally characterized, and the presence of a mutation in tumor tissue does not by itself prove that it is a causal driver of tumorigenesis.

The function of ATP synthase and the ETC is potentially altered by missense and nonsense mtDNA mutations, leading towards glycolysis by promoting a Warburg-like shift [30]. The mutation causes a decrease in activity, which produces a shift in the membrane potential of the mitochondria or the redox position of the electron carriers in the altered site, both downstream and upstream. These changes, however, may not deactivate oxidative phosphorylation but have the potential to alter the redox status of the ETC and ROS generation. Upstream of the chain, the shifting of the reduction state of electron carriers of complex V can be altered by mutation but cannot be abolished. This shift leads to a hike in superoxide generation from complex I, II, and III. This affects the ROS generation and changes the pattern of cytosolic redox signalling responsible for the regulation of cell proliferation, increasing the growth rate. These interlinked events by mtDNA lead to amplification of tumorogenesis. A study by Woo et al. [23] showed the link between tumorogenesis and stability of mtDNA, which is interplayed by increased ROS generation by mitochondria; however, the role of specific mtDNA mutations that regulate the phenotype of tumorogenesis is still unknown.

### 3.3 Therapeutic Implications of mtDNA-Driven ROS Dysregulation

Mitochondrial DNA mutations that partially impair respiratory complex activity, particularly within complexes I and III, frequently enhance superoxide generation and shift the redox state of upstream electron carriers without fully abolishing oxidative phosphorylation. Such mutations can amplify oncogenic signaling cascades (AKT or PI3K pathway activation) and promote a more oxidized cytosolic thiol environment, thereby fostering genomic instability, resistance to apoptosis, and metastatic dissemination. From a therapeutic perspective, these mtDNA alterations provide a rational basis to exploit mtROS as a cancer-selective vulnerability [31,32,33].

Tumors harboring a high burden of pathogenic mtDNA variants in ETC subunits or showing a strong dependence on oxidative phosphorylation for ATP and biosynthetic precursor production are expected to operate close to a critical redox threshold and may therefore be particularly susceptible to agents that further increase mtROS. In this context, pharmacologic inhibition of complexes I and II using mitochondria-targeted pro-oxidants (such as TPP^+^-conjugated derivatives that accumulate within the inner mitochondrial membrane) can accentuate electron leak, elevate superoxide and hydrogen peroxide production, dissipate mitochondrial membrane potential, and trigger regulated cell death (RCD) preferentially in malignant cells [34,35]. Conversely, neoplasms that maintain high OXPHOS flux while simultaneously upregulating antioxidant systems, including NRF2-driven transcriptional programs, glutathione metabolism, and thioredoxin/thioredoxin reductase networks, may buffer additional ROS and exhibit primary or acquired resistance to such pro-oxidant strategies. In these settings, rational combination regimens that pair complex I/II inhibitors or other mtROS-inducing agents with inhibitors of glutathione synthesis, thioredoxin reductase, or NRF2 signaling could be required to collapse mitochondrial redox homeostasis and fully unmask the cytotoxic potential of mtROS in OXPHOS addicted tumors [36,37].

However, these concepts are unlikely to apply uniformly to all cancers, and current data suggest that mtDNA-driven ROS dysregulation creates therapeutically exploitable vulnerabilities only in specific subsets of tumors defined by their mtDNA mutation burden, OXPHOS dependence, and antioxidant capacity, underscoring the need for careful functional validation and disease-specific stratification before broadly implementing such strategies [5,26].

## 4 Mitochondrial ROS in Tumor Progression and Metastasis

Once neoplastic transformation has been established, mtROS contribute to multiple hallmarks of tumor progression, including sustained proliferation, evasion of regulated cell death, metabolic cooperation with stromal cells, epithelial–mesenchymal transition (EMT), and metastatic dissemination [38]. Mitochondrial metabolism supports these processes by providing anabolic precursors, positioning mitochondria within regulated cell death signaling networks, and generating mtROS that modulate oncogenic pathways and redox balance under hypoxia and nutrient stress. Experimental models indicate that impairment of mitochondrial function or mtDNA depletion compromises tumor growth, whereas restoration of mitochondrial respiration and mtROS production can re-establish tumorigenic potential [39,40].

### 4.1 Requirement for Proliferation

Mitochondria are critical for the proliferation of cancer cells for obtaining adequate ATP from glycolysis unless pyruvate and uridine are supplied externally (to compensate for biosynthesis of aspartate and pyrimidine), as seen via *in vivo* experiments [41,42]. During tumor progression, cancer cells undergo metabolic reprogramming characterized by elevated glucose uptake and redistribution of carbon flux through the pentose phosphate pathway (PPP) [7], the Krebs cycle, and the ETC [5,6]. These adaptations are enabled by the reversibility of several TCA cycle reactions and the presence of multiple anaplerotic circuitries. Citrate remains an important intermediate operating as a major node of flexibility between catabolic and anabolic metabolism, which, besides acting as a fuel, gets converted to acetyl-CoA to export to the nucleus and cytoplasm. Cancer cells are in urgent need of the enzyme ACLY (ATP citrate lyase, which converts citrate to acetyl-CoA) for maximum proliferation rate [3]. The presence of a hypoxic environment and mitochondrial defects is the major source of citrate for the metabolism of reductive glutamine [4,43]. Elevated kinase activity of BRAF, leading to hyperactive MAPK signaling, is provoked by acetyl-CoA-derived acetoacetate, resulting in a hike in cell proliferation. Also, suppression of Phosphatase and Tensin Homolog (PTEN) or stabilization of Hypoxia-Inducible Factor 1-alpha (HIF-1α) by high ROS levels also initiates proliferation [38,44]. Further, mitochondrial dynamics and biogenesis, including metabolism, are controlled by the ROS level [45,46]. The production of ROS is increased due to the overexpression of ATPase inhibitory factor 1 (ATPIF1), which promotes the dimerization of ETC complex V to sequester ATP production as a side effect [47,48]. Proliferation is supported by ROS-driven cell death as it initiates the secretion of mitogenic factors acting on nearby cancer cells endowed with proliferative capacity [38].

### 4.2 Regulated Cell Death (RCD) Inhibition

Neoplasm transformation manifests in harsh cellular environments such as hypoxia, withdrawal of growth factors, and lack of nutrients, driving mitochondrial RCD through mitochondrial outer membrane permeabilization (MOMP) or mitochondrial permeability transition (MPT) [49,50]. Several alterations occur in malignant cells that increase the level of irreversible mitochondrial permeability, including the expression of B-cell lymphoma 2 (BCL2) proteins [51]. Some tumors, owing to high glycolytic rates and resistance to RCD, show high mitochondrial transmembrane potential whereby restoring the generation of pyruvate induces RCD and prevents tumor growth (*in vivo*) [30]. The first step of glycolysis, which is the conversion of glucose to glucose-6-phosphate in the presence of the enzyme HXK1 or HXK2 (hexokinase 1), removal of which caused unknown MOMP in cancer cells [52]. Glycolysis, oncogenic signaling, reduced glutamine carboxylation, ROS-mediated MPT, etc., are avoided by cancer cells by maintaining the best antioxidant defenses [53]. High resistance levels of cancer cells to MOMP and MPT can also be attributed to mitochondrial dynamics, as malignant cells tackle glucose deficiency via transition to oxidative phosphorylation on mitochondrial elongation [54]. This is helpful in generating a mitochondrial network due to mitophagic removal of nonfunctional components [55], which highlights the existence of a close and bidirectional link within mitochondrial RCD control and metabolism.

### 4.3 Stroma Interaction and Diversifications

Plasticity of a high degree, both in phenotypic and metabolic spheres, is marked in progressing malignancies due to the establishment of functional interactions with the non-transformed ones of the tumor environment [56,57]. Specifically, the tissue of origin strongly influences the metabolic dynamics of cancer cells [58,59]. Predominant reliance on glycolysis has been observed in CSCs from breast cancer, glioblastoma, and osteosarcoma, whereas ovarian cancer CSCs depend more on oxidative phosphorylation for ATP synthesis [60]. Because surface biomarkers for isolating CSCs are limited, mitochondrial metabolism itself may serve as a therapeutic entry point to characterize and target CSCs in different tumor types.

Preferential catabolism of glucose by subsets of CSCs of the same tumor [61,62], reprogramming for anaerobic glycolysis [63], increased alanine content [64], procurement of fatty acids from adipocytes [65,66,67] highlight the quasi-parasitic behavior of cancer cells in securing metabolic resources. This is due to the metabolic symbiosis observed in cancer cells from different regions of the same tumor in transporting lactate (derived from glycolysis) from regions of hypoxia to normoxic regions, intended to fuel oxidative phosphorylation so as to avoid competition for glucose [68,69].

### 4.4 Release of Metastasis Cascades

EMT is one of the earliest and most critical changes that increases the invasiveness of cancer cells, and several mitochondrial metabolites, particularly fumarate, contribute to this process [70,71]. Successful metastasis dispersion requires oxidative phosphorylation and good biogenesis of mitochondria [72,73]. The changes in the cytoskeleton structure of cancer cells critical for their motility in local invasion are sponsored by oxidative metabolism of mitochondria [74,75]. ROS overproduction is moderately promoted by mitophagic abnormalities, which switch on many signaling pathways involved in metastasis, such as protein tyrosine kinase 2 beta and SRC [76,77]. It has been found that dysfunction of mitochondria is evident in patients with tumors of as many as nine different types, which is also associated with ROS overproduction [45,78]. Oxidative stress and ROS production remain the preliminary consequences for metastasis, followed by an increase in glucose uptake, which may differ according to the anatomical sites [79,80]. This underlies the explicit role of mitochondria in the spread of cancer malignancies.

### 4.5 Mitochondrial Calcium in Metabolism of Cancer Cells

Cell growth and metabolism are linked to mitochondrial Ca^2+^ signaling, which is a prosurvival mechanism by activation of multiple components of the TCA cycle, which ultimately feeds the ETC chain for ATP production [81]. The fundamental machinery for inducing apoptosis is dependent on this calcium signaling pathway, which occurs by opening the mPTP (mitochondrial permeability transition pore) and release of cytochrome c [82,83]. Specialized domains such as MAMs (mitochondria-associated membranes) are involved in connecting the mitochondria to the ER (endoplasmic reticulum), which remains the storehouse of intracellular calcium. Many tumor suppressors that stay in the MAMs are capable of modulating the Ca^2+^-ER-mitochondria relation, which regulates cell death post exposure to cancer therapies [84]. A few studies have shown the intrinsic role of mitochondrial calcium in changing the energy status of cancer cells. The studies have found that there is an aberrant expression of the MCU (mitochondrial calcium uniporter) complex, which has drawn a complex scenario [85]. This MCU-dependent mitochondrial Ca^2+^ increase has been found to be associated with cancer invasion, metastasis, and low prognosis, which occurs by upregulating the activity of the TCA cycle and NADH/NAD^+^ ratio [86]. An increase in mitochondrial respiration during the course of mitosis is observed in MCU activation mediated by AMPK, which also allows a speedy transit of mitochondrial Ca^2+^ [87].

In hepatocellular carcinoma and breast cancer, it was seen that downregulation of MICU1 (mitochondrial calcium uptake 1) negatively regulates MCU and works as a gatekeeper for mitochondrial Ca^2+^, showing poor prognosis [88]. Also, AKT-promoted MICU1 instability is related to high levels of Ca^2+^ intake, high ROS production, and tumor progression [89,90]. The inhibition induced by overexpression of MICU1 is taken as an advantage by cancer cells rather than activation of the mitochondrial Ca^2+^ storage, which leads to a glycolysis drive for ATP production [91]. Recent studies show that the amount of released Ca^2+^ is related to the tumor suppressor BAP1 from the endoplasmic reticulum into the mitochondria and cytosol, thereby increasing apoptosis and glycolysis [91]. A key event that characterizes various malignancies and their resistance is the regulation of the autophagic pathway due to the correlation of mitochondrial matrix Ca^2+^ entry and biogenesis. Tumor suppressors such as Promyelocytic Leukemia (PML) and p53 are essential for suppressing autophagy needed for cancer development by maintaining optimal ER-Ca^2+^ transfer. It has been observed that a low level of ATP production, high resistance to metabolic stress, and activation of autophagy by AMPK in cancer cell downregulated with PML and p53 [92]. The requirement of basal mitochondrial Ca^2+^ is critical for cancer cells, and inhibitors of ER-mitochondria Ca^2+^ decrease the oxophosphorylation, which induces autophagy [93]. This shows that autophagy and ER-Ca^2+^ flux are correlated, which may turn into for Ca^2+^ based treatment.

Recent studies have highlighted the critical role of ROS-mediated calpain activation in cancer progression, metastasis, and therapeutic response. Emerging evidence demonstrates that mtROS accumulation induces calcium dysregulation and activates calpain-dependent signaling pathways involved in Nuclear Factor Kappa-light-chain-enhancer of activated B cells (NF-κB) activation, HIF-1α stabilization, mitochondrial dysfunction, and CSCs survival [94,95,96]. Moderate ROS levels may promote tumor adaptation and progression, whereas excessive ROS accumulation induces ER stress, mitochondrial damage, and apoptotic or pyroptotic cell death [97]. Furthermore, ROS-induced calpain activation contributes to metabolic reprogramming, therapy resistance, and tumor microenvironment remodeling, emphasizing the context-dependent role of redox signaling in cancer biology [94].

Recent therapeutic investigations suggest that targeting the ROS calpain axis may offer promising opportunities for precision oncology. Several ROS-generating chemotherapeutics, natural compounds, and calpain inhibitors have shown potential to induce oxidative stress-mediated cancer cell death through mitochondrial dysfunction and calcium-dependent apoptotic signaling [98]. In addition, modulation of mtROS and calpain activity may improve therapeutic sensitivity while limiting resistance mechanisms in aggressive tumors and CSCs [94,99]. Collectively, these findings support the ROS calpain signaling network as a promising therapeutic target for future mitochondrial-directed anticancer strategies.

### 4.6 Mitochondria-Associated Membranes (MAMs), ROS, and Metastatic Signaling

MAMs are enriched in oncogenes and tumor suppressors localized at the ER–mitochondrial interface, which can modulate MAM function and alter cell death pathways [100]. Recent studies have shown that MAMs are central hubs in key cellular signaling pathways, and their dysfunction plays a major role in many cancers. Among oncogenes, AKT is physically and functionally linked to MAMs. AKT phosphorylates inositol 1,4,5-trisphosphate receptor type 3 (IP3R3), thereby inhibiting ER Ca^2^^+^ release and apoptosis [101]. It also phosphorylates hexokinase 2, increasing its association with MAM protein VDAC1 and preventing Ca^2^^+^-dependent apoptotic responses [102].

VDAC1 is associated with MAMs and coordinates the exchange of metabolites, ions, and Ca^2^^+^ between mitochondria and the rest of the cell [103]. It is overexpressed in many cancers and, together with HK-I and HK-II, is also overexpressed, which helps in the detachment of HK from the binding site of the mitochondria by peptide VDAC1-based [104]. Along with the association with cancer-related proteins, MAMs have a critical role in controlling cell death due to the presence of many tumor suppressors, of which PTEN remains the most common. In the MAM, it interacts with IP3R3 and regulates the Ca^2^^+^ release from the ER [105]. Thus, MAMs have major implications for Ca^2^^+^ signaling and are critically important for cellular metabolism and survival.

## 5 Pro-Tumor and Anti-Tumor Functions of Mitochondrial ROS

mtROS occupy a paradoxical position in cancer biology: at low to moderate levels they act as critical second messengers that sustain oncogenic signaling, metabolic reprogramming, and microenvironmental adaptation, whereas at higher, poorly buffered levels they inflict lethal damage and activate RCD pathways [2,6]. This threshold-dependent behavior underlies both the pro-tumorigenic and anti-tumorigenic effects of mtROS and explains why they can promote tumor growth in some contexts while constraining it in others. In this section, we separate the signaling pathways and cellular processes in which mtROS support tumor fitness from those in which they limit tumor growth or sensitize cancer cells to therapy.

ROS are increasingly recognized as key mediators of cellular signaling with a dual role in cancer progression [18]. On the one hand, they enhance protumorigenic signaling that promotes cancer cell proliferation, survival, and adaptation to hypoxia; on the other hand, excessive ROS can trigger antitumorigenic pathways leading to oxidative stress-induced cell death. Cancer cells increase their ROS production rate to hyperactivate the cellular signaling required for cellular transformations and tumorigenesis. To maintain ROS homeostasis and interfere with the cell death process, cancer cells increase their antioxidant activity. As compared to normal cells, these disturbances in the redox environment within the cancer cells may lead to increased response to ROS-based therapies.

### 5.1 Sourcing ROS

Signalling-based ROS are produced mainly by membrane-bound NOX and mitochondria. In cellular respiration, a series of mitochondrial complexes passes the electrons to reach the end acceptor complex (O_2_), wherein sometimes electrons may leak to react with O_2_, forming O_2_^−^. Of all the ten sites of O_2_^−^ production, mitochondrial complexes such as I, II, and III are involved in redox signaling [106]. Both intracellular and extracellular production of O_2_^−^ from O_2_ and NADPH are catalyzed by NOXs. Although found in other membranes such as the endoplasmic reticulum, nucleus, mitochondria, etc., the primary location of NOXs is the plasma membrane. The most critical aspect of both NOXs and mitochondria is that both can spatially localize towards oxidant acceptors to relay ROS-mediated signaling. However, with the increase of excessive levels of ROS, H_2_O_2_ diffuses away to distant places of the cell, inducing cell death and oxidative stress. Thus, factors such as ROS type, antioxidant amount, and concentration level determines ROS is toxic or signaling in nature [107].

### 5.2 ROS Regulation

Optimal cell signaling and normal cell survival require the homeostasis of ROS. Signaling pathways regulating differentiation, metabolic adaptation, and cellular proliferation are activated by low levels of ROS [108]. Spatially localized ROS are present in higher levels in cancer cells compared to normal cells, which elevates activation of cellular signaling critical for the transformation of cells and tumorigenesis. This accumulation of ROS occurs either due to increased production or decreased ROS elimination [109]. ROS increases are promoted due to activation of oncogenes, increased metabolism, and hypoxia, and the loss of tumor suppressors can minimize ROS elimination, but the accumulation of ROS must be regulated to prevent cancer [110]. ROS homeostasis is maintained by cells through regulating intracellular levels of ROS in a temporal and spatial manner using their antioxidant defense system. O_2_^−^ is capable of damaging and causing inactivation of iron-sulfur cluster-containing proteins. Various cellular components contain SOD1 for the rapid conversion of O_2_^−^ to H_2_O_2_. Many antioxidants exist to convert intracellular H_2_O_2_ to H_2_O, thus preventing cellular toxicity and maintaining the optimal level of H_2_O_2_ for cell signaling. These include antioxidants such as catalase (CAT), peroxiredoxins (PRXs), and GPx. Hence, a robust antioxidant mechanism at the cellular level regulates the ROS level.

### 5.3 Promotion of Pro-Tumorigenic Signals

Early studies have shown that ROS promotes tumor growth, and H_2_O_2_ activation is induced by oncogenic Ras due to the activation of the PI3K/AKT/mToR and MAPK/ERK signaling pathways by growth factors. The PI3K/AKT/mTOR pathway is hyperactivated by ROS via oxidizing and inactivating the phosphatases, and this pathway is found to be activated in many cancers. ROS production can further be increased by the oncogenic activation of AKT, promoting cancer cell survival and proliferation [111]. MAPK phosphatases are also inactivated and oxidized by ROS, which also includes activation of growth factor receptors and pro-proliferative signaling of MAPK/ERK [112,113]. mtROS regulates the anchorage-independent lung cancer growth via the MAPK/ERK pathway induced by *Kras*, and complete disruption of the mitochondrial chain showed a diminishing effect in tumorigenesis [17].

Tumor survival is also promoted by ROS by activating NRF2 and NF-κB, including other transcription factors that hyperactivate the expression of antioxidants to attack the ROS-dependent cancer cell death [114]. Metastasis and angiogenesis in tumors are promoted by ROS, which are interrelated processes leading to poor prognosis. When the blood supply in highly proliferative tumors is outgrown, ROS production in regions of the solid tumors is elevated as those regions become hypoxic and glucose-deprived. Metabolic changes become inevitable in such a tough microenvironment for cell survival and proliferation, which includes activation of Adenosine Monophosphate (AMP)-activated protein kinase (AMPK) and stabilization of HIF and the metabolism of the one-carbon pathway to enhance the production of NADPH to maintain the redox balance [115].

### 5.4 Promotion of Antitumor Signaling

**Figure 1:**
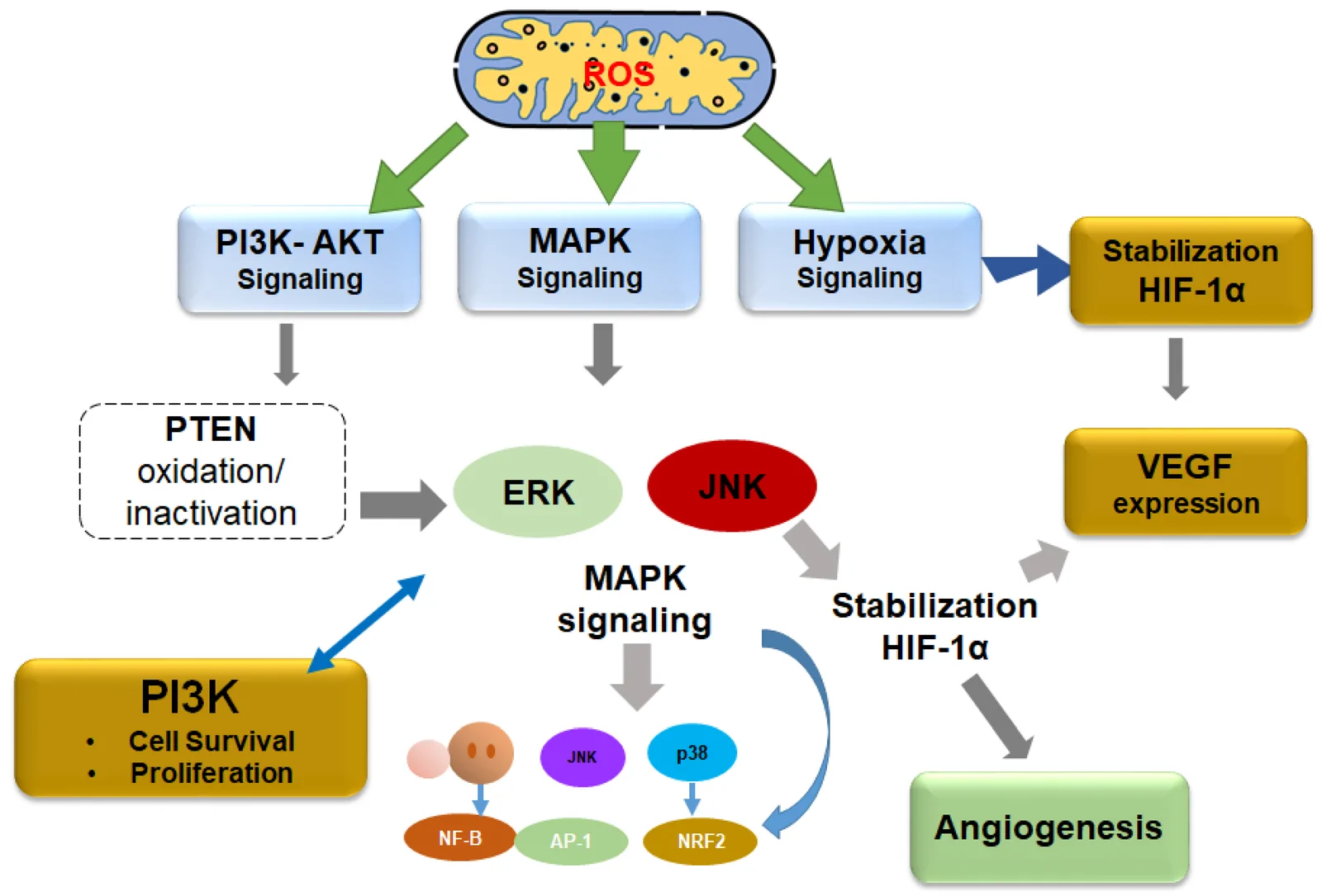
**ROS-mediated oncogenic signaling pathways in cancer.** mtROS act as critical signaling molecules that regulate several oncogenic pathways involved in cancer progression. ROS can oxidize and inactivate the tumor suppressor PTEN, resulting in activation of the PI3K/AKT signaling pathway that promotes cell survival and proliferation. Additionally, ROS stimulates the MAPK cascade, including ERK, JNK, and p38 pathways, which regulate tumor growth, migration, and stress responses. mtROS also stabilizes HIF-1α, leading to increased expression of angiogenic factors such as vascular endothelial growth factor (VEGF). These pathways activate downstream transcription factors, including NF-κB, AP-1, and NRF2, collectively contributing to tumor growth, angiogenesis, and metastatic progression.

Senescence, cell cycle arrest, and cancer cell death may occur when the ROS levels are too high, which also happens due to the activation of signaling pathways such as ASK1/p38 and ASK1/JNK [116]. The level of ROS increases, and the amount of reduced GSH decreases when tumor cells detach from the extracellular matrix to invade the basement membrane. The survival and proliferation of circulating cancer cells are prevented by the oxidizing environment of the blood, and thus, high ROS levels prevent metastasis at distant [117]. Thus, a possible therapeutic pathway using ROS is the perturbation of the ROS-dependent pathways (Fig. 1).

### 5.5 Exploiting the Dual Role of ROS for Therapy

The dual pro-tumorigenic and antitumorigenic properties of ROS highlight the importance of tailoring redox-based interventions to the intrinsic oxidative status and adaptive capacity of individual tumors. At low to moderate levels, mtROS act as signaling second messengers that support proliferation, EMT, angiogenesis, and immune evasion, whereas excessive or unbuffered ROS cause oxidative damage to macromolecules and organelles, culminating in diverse forms of RCD [118]. Therefore, therapeutic strategies that deliberately push mtROS above the survival threshold in cancer cells by enhancing electron leak at respiratory complexes, disrupting electron carriers, or blocking key antioxidant defenses can preferentially eliminate malignant cells that already operate under chronic oxidative stress while sparing normal cells with lower basal ROS and more robust redox buffering capacity [119].

Pro-oxidant approaches are likely to be most effective in tumors characterized by high metabolic rates, OXPHOS dependence, and limited antioxidant reserve, where further increases in mtROS rapidly trigger mitochondrial permeability transition, cytochrome c release, and cell death. In contrast, in tumors or microenvironmental niches in which ROS-driven signaling primarily fuels tumor-promoting inflammation, stromal activation, or immune dysfunction, carefully titrated mitochondria-targeted antioxidants or redox-modulating combinations may be advantageous [35,120]. Mitochondria-targeted antioxidants such as MitoQ or MitoTEMPO, as well as emerging nanomaterial-based redox modulators, have the potential to normalize aberrant mtROS signaling, protect non-malignant tissues from therapy-induced oxidative injury, and improve the functionality of antitumor immune cells when integrated thoughtfully with chemotherapy, radiotherapy, or immune checkpoint blockade [37,121,122,123]. Collectively, these observations argue for the development of redox-stratified treatment algorithms in which quantitative assessment of tumor- and host-derived ROS, together with profiling of mitochondrial antioxidant capacity, guides the selection between pro-oxidant and antioxidant-leaning mtROS targeted interventions.

### 5.6 Pro-Tumorigenic mtROS Signaling

The proliferative phenotype of cancer and oncogenic transformations are associated with a change in thiol redox balance (cytosolic), leading to a more oxidized state [124]. Genomic instability is caused by such shifts, as ROS present in the cytosol may enter the nucleus during DNA replication, causing mutations. Low levels of mtROS are important for the regulation of protein function and signaling. Targets of this nature include cysteine groups (reactive) on proteins, which may be oxidized by H2O2 but not by superoxide. Functional and conformational changes are caused by the oxidation of thiols, which may also form both intra- and inter-molecular dithiol linkages. Generally, lipid and protein phosphatases are more prone to ROS attack, where oxidation leads to their inactivation [125,126]. Kinase pathways such as AKT and MAPK-ERK pathways involved in cancer cell growth and proliferation are influenced heavily by oxidative inactivation of phosphatases. It is very much possible for tumour cells to mimic the effects of genetic mutations in cells so as to continuously increase the generation of basal ROS [127].

### 5.7 ROS Role in Killing Cancer Cells

#### 5.7.1 ROS in Apoptosis

ROS induces all forms of DNA damage, such as strand breakages, base modifications, and DNA-cross linking, which are linked to cancer initiation and progression [128]. Many studies have shown that anticancer agents promote cancer cell autophagy and apoptosis due to ROS production, as shown in Table 1.

**Table 1:** Cancer cell death caused by elevated levels of ROS.

Cell Mechanisms	Consequences
Elevation of oxidation via the c-Met-Nrf2-HO-1 pathway [129]	Apoptosis
ROS increases apoptosis through Protein Kinase B (AKT) and Mitogen-Activated Protein Kinase (MAPK), and DNA damage promotes p53 [130]	
Decreases Reactive Oxygen Species (ROS) production by expression of Glutathione Peroxidase 3 (GPx3), which leads to G2/M arrest [131]	
Increase in ROS via knockdown of nicotinamide nucleotide transhydrogenase and apoptosis in oxidative stress [132]	
Apoptosis is induced through the regulation of miR-21 and the stress pathway ROS/ER [133]	Short mRNA
miRNA biogenesis regulation through disruption of ROS-dependent MITF-DICER pathway [134]	
Using siRNA technology NADPH Oxidase 2 (NOX2) can be downregulated [135]	
Activation of MAPK pathway members such as JNK and Extracellular Signal-Regulated Kinase 1/2 (ERK1/2) induces cell killing [136]	Autophagy
Enhanced autophagy due to Yes-Associated Protein (YAP) silencing by elevated RAC1-induced ROS by inactivating mTOR [137]	
Autophagy is induced by Zinc Oxide Nanoparticles and ROS generation [138]	

#### 5.7.2 ROS in Autophagy

Increased ROS levels lead to autophagy, which involves the degradation of proteins and organelles, as shown by studies [139]. The effects of autophagy range from infection prevention to the removal of pathogens, cell death, and the removal of nonfunctional cellular organelles. These functions show that ROS has the potential to act as a signaling target related to survival in autophagy [140]. In malignant tumors, induction of autophagy is regulated by ROS [141].

#### 5.7.3 ROS and microRNAs (miRNAs)

ROS and miRNAs are both dysregulated in cancer, and they promote tumorigenic conversion through their association and by maintaining homeostasis through crosstalk between them. miRNAs act as essential targets for ROS-mediated stress molecules; they also control genes that are ROS activators and scavengers. Studies have shown that miR-34a targets *NOX2* genes, inducing apoptosis in glioma cells via *NOX2*-derived ROS generation [142]. In a similar fashion, miR-23b downregulates the expression of proline oxidase by 3′UTR targeting, which promotes renal cancer [143]. Thus, ROS and miRNAs are interlinked and play a critical role in cancer progression, and targeting ROS via miRNA inhibitors may be a novel therapeutic approach.

## 6 Mitochondrial ROS in Therapy Response, Resistance and Immunosurveillance

Many anticancer therapies, including chemotherapy, radiotherapy, and targeted agents, engage mtROS as part of their cytotoxic mechanisms, but the same mitochondrial redox circuitry can promote resistance and shape antitumor immunity [26]. Therapy-induced increases in mtROS may push cancer cells beyond critical bioenergetic and redox thresholds, triggering regulated cell death, yet tumors often adapt by reprogramming mitochondrial metabolism and upregulating antioxidant defenses. In parallel, mtROS in tumor and immune cells modulate antigen presentation, checkpoint expression, effector T-cell function, and myeloid cell polarization, thereby influencing the balance between antitumor and protumor immunity [1].

### 6.1 mtROS in Therapy Response and Resistance

Permanent inactivation or death remains the primary objective for radiation therapy, chemotherapeutics, or targeted anti-cancer agents [144]. The phenomenon of RCD, which is initiated by these therapies, is controlled by the involvement of mitochondria, which stresses them to undergo MPT or MOMP, leading to resistance [145]. The regulation of necrotic or apoptotic RCD involves factors such as: 1) robust metabolic component; 2) influence in therapy due to mitochondrial biology; 3) Elevated levels of formation of anti-cancer agents by metabolic enzymes within the mitochondria [146]. For example, melanoma cells resist therapy by switching from glycolysis to OXPHOS for the production of energy in BRAF^V600E^ inhibition by vemurafinib [147]. Also, a switch from glucose to lactate is observed in breast cancer cells in resistance to PI3K inhibition, which serves as a source of carbon units [148]. Hence, compensatory metabolic networks are established by different treatments, which promote the process of cancer cell survival, and metabolic reconnection by mitochondria has a huge impact on responses shown by cancer cells during therapy.

### 6.2 mtROS in Tumor Cell Intrinsic Immune Evasion

Within tumor cells, mtROS can influence immunosurveillance by modulating antigen presentation, expression of immune checkpoints, and secretion of immunomodulatory factors. Elevated mtROS may promote neoantigen generation through increased genomic instability, yet chronic oxidative stress can also favor PD-L1 upregulation and release of immunosuppressive cytokines and metabolites, such as lactate, that impair effector T-cell function [1,8]. mtROS and mtDNA release also intersect with cytosolic DNA sensing pathways (e.g., cGAS–STING), which can drive type I interferon responses and antitumor immunity in some contexts but may become dampened as tumors adapt.

Immunosurveillance is influenced by mitochondria by both intrinsic and extrinsic cancer cell mechanisms. With the death of cancer cells, they release danger signals that are stored in mitochondria, which are important for the activation of dendritic cells to smoothly carry out an immune response targeting tumor cells [149]. Many functions related to anticancer immunity are carried out by mitochondrial metabolism, such as differentiation and tumoricidal activity of certain macrophages, activation of the inflammasome, and immunological memory formation [150]. ATP remains the best-characterized product that promotes immune responses to dying cancer cells. These cells can release extracellular ATP in considerable amounts only if they can initiate autophagic responses prior to death [151]. They are capable of mediating important chemotactic and immunostimulatory functions when binds to purinergic receptor P2X7 (P2RX7) and purinergic receptor P2Y2 (P2RY2) on the surface of dendritic cells or their precursors. Those autophagic malignant cells that lost their ability to propagate anticancer immunity due to therapy can be revived by inhibiting the degradation of ATP by CD39 (ectonucleoside triphosphate diphosphohydrolase 1; ENTPD1) [152].

### 6.3 mtROS in Immune Cells and Antitumor Immunity

In immune cells, mtROS play distinct, cell-type-specific roles that are critical for effective antitumor responses. In T cells, controlled mtROS bursts support activation, differentiation, and effector function, whereas chronic or excessive mtROS in exhausted, nutrient-deprived T cells can impair mitochondrial fitness and exacerbate dysfunction [26]. Dendritic cells rely on mitochondrial metabolism and ROS for antigen processing and cross-presentation, but overwhelming oxidative stress may limit their ability to prime cytotoxic T lymphocytes. In macrophages and other myeloid cells, mtROS contribute to polarization toward pro-inflammatory or immunosuppressive states, influencing cytokine profiles and the balance between tumor-restraining and tumor-promoting myeloid populations [1].

## 7 Therapeutic Targeting of Mitochondrial ROS: Opportunities and Challenges

The central role of mitochondria in cancer metabolism and redox signaling has motivated the development of therapeutic strategies that either amplify mtROS to drive cancer-selective cell death or dampen mtROS to protect normal tissues and immune cells [5]. Pharmacologic approaches include inhibition of respiratory complexes, disruption of mitochondrial antioxidant systems, and the use of mitochondria-targeted pro-oxidant or antioxidant compounds and nanoplatforms, many of which show promising preclinical activity but more variable clinical performance [4]. In the following sections, we summarize major classes of mitochondria-targeted agents, how they modulate mtROS, and the opportunities and limitations of exploiting mtROS as a therapeutic vulnerability in oncology.

### 7.1 Targeting Mitochondrial Biochemical Functions

Multiple carbon fuels are used by mitochondria to produce metabolites and ATP generated from glycolysis and amino acids, such as fatty acids and glutamine. These fuels are then fed to the TCA, producing reducing equivalents such as NADH and FADH2, which supply electrons to the ETC [153]. Hydrogen ions are consecutively added by the ETC from the matrix to the inner membrane of complexes I, III, and IV. Intermediates are generated from the TCA cycle that relay various biosynthetic pathways for producing lipids, glucose, amino acids, nucleotides, and heme. Thus, mitochondria act as a central node for both catabolic and anabolic metabolism.

### 7.2 Signaling Organelles of Mitochondria

The metabolic demands of a cell are fulfilled by changing the biogenetic and biosynthetic functions, and continuous communication is made by mitochondria to ensure the fitness of the cell. It has been proposed that when proliferation is about to begin, which is a metabolically demanding factor, a checkpoint of internal nature is made prior to transcription [154]. Anterograde and retrograde signaling are the two types of communication followed by the mitochondria. Anterograde signaling deals with cytosolic signaling by sequestering calcium to the mitochondrial matrix in the presence of elevated levels of cytosolic calcium. This activates the TCA cycle enzymes to increase the oxidative metabolism of mitochondria. On the other hand, signal transduction from mitochondria to cytosol is retrograde signaling, which was added by the discovery of cytochrome c. Recently, many retrograde mechanisms have been discovered for the release of ROS and metabolites. Oxaloacetate and acetyl-CoA are produced by metabolites such as citrate in the cytosol and cleaved by ATP citrate lyase [155]. Protein acetylation is regulated by acetyl-CoA, which alters protein activity. Thus, mitochondria, acting as a signaling organelle, are supported by various findings such as phosphatases and kinases expression in the mitochondrial matrix, multiple modes of signaling, and the presence of a signaling platform in the outer membrane of the mitochondrial ETC [156,157].

### 7.3 Tumor Growth Requires Mitochondrial Metabolism

Tumor cells are actively involved in glycolysis and mitochondrial metabolism, providing the basic blocks such as lipids, amino acids, and nucleotides synthesis critical for cellular proliferation. A high glycolysis rate results from dysregulation of signaling pathways such as PI3K and subsequent activation of oncogenes such as KRAS and MYC. This incident leads to the generation of glycolytic intermediates, which then transport themselves to various other pathways involved in cellular proliferation, such as the pentose phosphate pathway for the production of NADPH and nucleotides [158]. Generation of ATP and intermediates of the TCA cycle are elevated due to an increase in mitochondrial metabolism, which are used as precursors for the synthesis of macromolecules [159]. Due to the oxidative metabolism occurring in cancer cells, a huge amount of ROS is produced in the ETC of mitochondria, which activates signaling pathways near the mitochondria that initiate cancer cell proliferation and tumor progression [110]. Since the accumulation of ROS leads to cell death, cancer cells generate a huge amount of NADPH both in the cytosol and mitochondria to compensate for the high antioxidant activity and to prevent the ROS buildup. Thus, mitochondrial metabolism, along with glucose metabolism, is essential for tumor cell propagation.

### 7.4 Direct Pharmacologic Targeting of mtROS

Cancer cells typically maintain elevated basal levels of mt ROS as a consequence of oncogene activation, mtDNA mutations, and altered ETC activity, positioning at least some malignant clones closer to a redox threshold beyond which additional oxidative stress becomes cytotoxic [5]. However, the magnitude and even the existence of such a “therapeutic window” are highly context-dependent and influenced by factors such as intratumoral heterogeneity in OXPHOS dependence, variability in antioxidant capacity, and the relative sensitivity of surrounding normal tissues to mtROS perturbation [4]. Thus, although pharmacologic agents that further increase mtROS or selectively disrupt mitochondrial antioxidant defenses can, under favorable conditions, preferentially kill subsets of malignant cells, this selectivity cannot be assumed a priori and must be empirically established for each disease context.

#### 7.4.1 Complex I/II Inhibition and TPP^+^-Conjugated Pro-Oxidants

Several mitochondria-targeted small molecules exploit the generation of mtROS at respiratory complexes I and II to induce cancer-selective cell death. Agents such as IACS-010759 and MitoTam are potent complex I inhibitors that suppress OXPHOS, promote a compensatory shift toward glycolysis, and concomitantly increase mtROS production within the inner mitochondrial membrane. In preclinical models, IACS-010759 and related complex I inhibitors show strong antitumor activity in OXPHOS-dependent leukemias and solid tumors, particularly when combined with agents that limit glycolytic compensation [5]. In clinical model, MitoTam a tamoxifen derivative conjugated to the lipophilic triphenylphosphonium (TPP^+^) cation, accumulates in mitochondria, blocks ubiquinone binding at complex I, elevates ROS, collapses mitochondrial membrane potential, and triggers apoptosis and necroptosis; in a phase I/Ib trial, it achieved durable disease control particularly in renal cell carcinoma patients, consistent with high renal accumulation of the drug [121,160]. Similarly, mitochondria-targeted vitamin E succinate analogues such as MitoVES and related TPP^+^-conjugated compounds localize to the inner mitochondrial membrane, inhibit complex II, enhance superoxide generation, and have shown robust pro-apoptotic and antitumor activity in multiple preclinical tumor models [161]. These findings support the concept that controlled pharmacologic amplification of mtROS at respiratory complexes can be harnessed as a cancer-selective killing strategy.

#### 7.4.2 Mitochondria Targeted Antioxidants and Redox Buffering Strategies

In parallel to pro-oxidant approaches, mitochondria-targeted antioxidants such as MitoQ and MitoTEMPO have been developed to selectively scavenge mtROS in preclinical models. These agents consist of antioxidant moieties (e.g., ubiquinone or TEMPO) linked to a TPP^+^ cation that drives their accumulation within the mitochondrial matrix, where they can limit oxidative damage to mitochondrial DNA, lipids, and respiratory complexes. However, preclinical studies in BRAF-driven melanoma and KRAS-driven lung cancer models have shown that MitoQ and MitoTEMPO do not reduce primary tumor burden or metastasis when used as monotherapies, although they can alter cancer cell bioenergetics and extracellular acidification rates, suggesting complex context-dependent effects on tumor progression [162,163]. These results indicate that mitochondria-targeted antioxidants may be more suitable as adjuvants to protect normal tissues, to prevent therapy-induced toxicity, or to reshape the tumor microenvironment rather than as stand-alone cytotoxic anticancer agents, and they highlight the importance of carefully defining disease context and endpoints when designing mtROS scavenging strategies [164].

#### 7.4.3 Nanomaterial-Based Modulation of mtROS

Recent advances in nanotechnology have enabled the design of smart nanomaterials that preferentially accumulate in mitochondria and either amplify or deplete mtROS in a spatiotemporally controlled manner. Functionalization of nanoparticles with lipophilic cations, peptide targeting sequences, or mitochondrial penetrating ligands allows precise delivery of ROS-generating payloads (such as photosensitizers, chemotherapeutics, or redox cycling agents) directly to the mitochondrial compartment, thereby maximizing local oxidative damage while reducing off-target toxicity [165]. Conversely, mitochondria-targeted nanocarriers loaded with antioxidant enzymes or small molecule scavengers can buffer excessive mtROS to protect healthy tissues or immune cells during aggressive therapy. These nanoplatforms offer the possibility of maintaining mtROS levels within tumors, integrating diagnostic imaging with therapy (theranostics), and overcoming limitations of small molecule distribution, although their clinical translation will require rigorous evaluation of long-term safety, pharmacokinetics, and immunogenicity [123].

#### 7.4.4 Context Dependence and Clinical Limitations

Despite strong mechanistic rationale and encouraging preclinical data, the clinical translation of mtROS-targeted therapies has been mixed, underscoring that mtROS do not represent a universal Achilles heel across all cancers [5]. Tumors display substantial heterogeneity in mitochondrial content, OXPHOS dependence, basal ROS levels, and antioxidant buffering, not only between tumor types but also between regions and clones within the same lesion, which can create ROS-insensitive subpopulations that survive pro-oxidant stress [4]. Metabolic plasticity constitutes a second major limitation. Cancer cells can dynamically rewire fuel utilization and redox metabolism, shifting between glycolysis and OXPHOS, increasing reliance on glutamine, lactate, or fatty acids, and upregulating alternative NADPH pathways to restore redox balance after ROS-targeted interventions [3,6]. Such adaptive rewiring has been implicated in resistance to OXPHOS inhibitors and kinase inhibitors whose efficacy depends on mtROS-driven cell death pathways. Many tumors robustly induce antioxidant programs in response to therapy. Constitutive or therapy-induced activation of NRF2, inactivation of KEAP1, and increased expression of GSH, thioredoxin, and peroxiredoxin systems allow malignant cells to buffer additional mtROS and blunt the cytotoxic impact of pro-oxidant drugs, while simultaneously promoting resistance to conventional chemotherapy and radiotherapy [26]. These adaptive antioxidant responses reduce the effective width of the putative therapeutic window. Finally, normal tissues and immune cells may be injured by systemic mtROS modulation, and several mitochondria-targeted agents and broader mitochondrial metabolism drugs have shown limited efficacy or dose-limiting toxicity in early-phase trials, highlighting issues of drug distribution, target engagement, and on-target side effects [5]. Together, these observations argue that mtROS-directed therapies require careful patient stratification, dosing strategies that minimize normal tissue damage, and rational combinations with agents that constrain metabolic and redox adaptation.

### 7.5 Targeting mtROS-Ca^2^^+^ Crosstalk and MAMs

As discussed in Section 4.5 and Section 4.6, mitochondrial Ca^2^^+^ handling and mitochondria-associated membranes (MAMs) critically shape mtROS production, permeability transition, and cell death sensitivity, making the ROS–Ca^2^^+^–MAM axis an attractive but highly context-dependent therapeutic target.

The progress of cancer therapy is often limited by tumor heterogeneity and the existence of compensatory pathways. As summarized in Table 2, many drugs target different mitochondrial mechanisms, and several studies suggest that simultaneously inhibiting multiple mitochondrial pathways can yield stronger antitumor effects than single-agent approaches [166]. Because mitochondria are central hubs for cellular communication, metabolism, and redox signaling, they represent critical but context-dependent therapeutic targets. Autophagy, which can confer resistance to mitochondrial stress, is one such adaptive mechanism; combining autophagy inhibitors with standard therapies has shown promising results in preclinical and early clinical studies. In addition, targeting the PI3K/Akt/mTOR axis can enhance chemosensitivity and counteract mitochondrial reprogramming that otherwise limits the efficacy of mitochondrial drugs.

**Figure 2:**
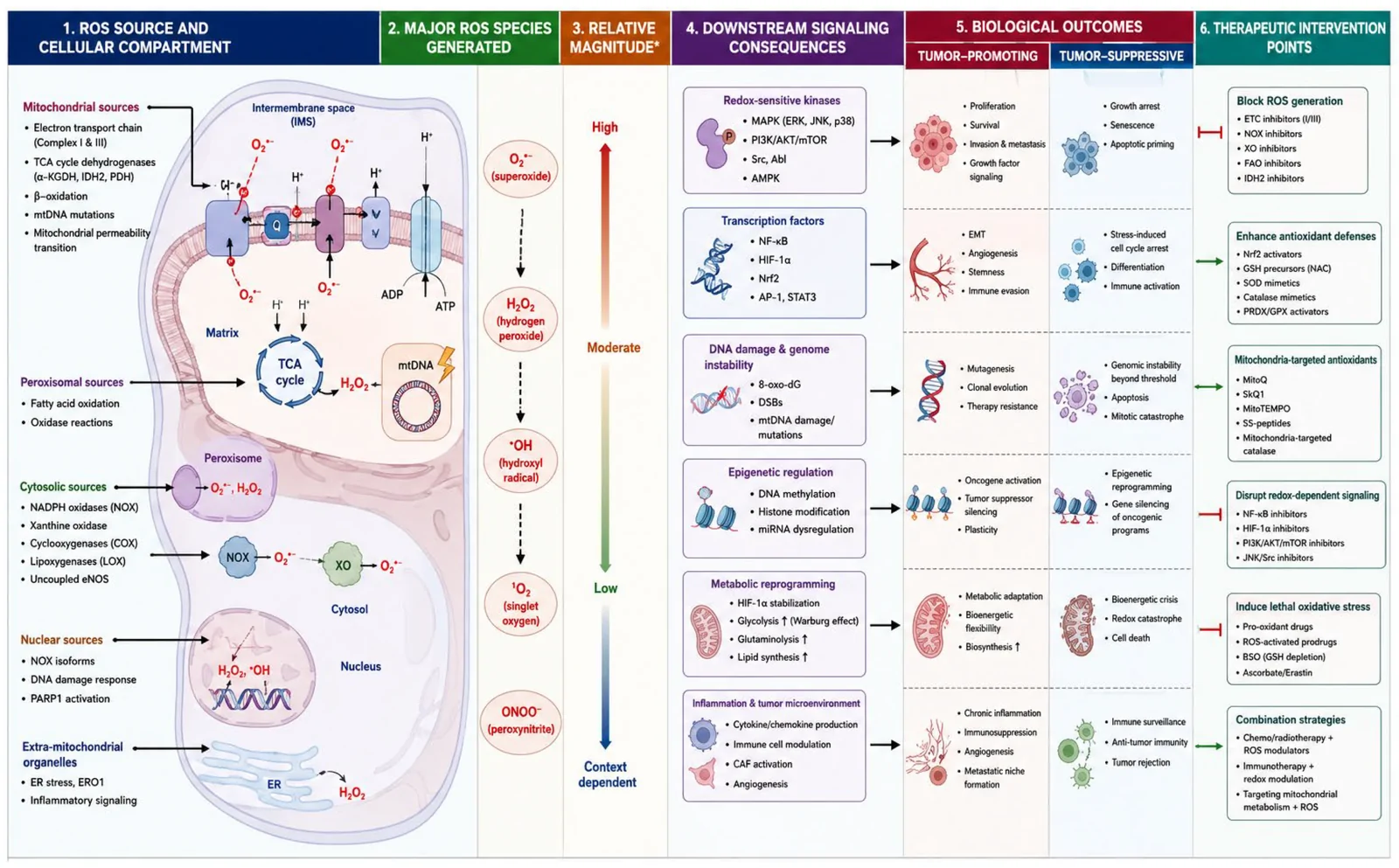
**Schematic of ROS sources, signaling, biological outcomes, and therapeutic strategies in cancer.** Major intracellular ROS sources include the mitochondrial ETC, peroxisomes, NOX, endoplasmic reticulum, cytosol, and nucleus. Key ROS species (superoxide, hydrogen peroxide, hydroxyl radical, singlet oxygen, peroxynitrite) are shown with their relative abundance and compartmental distribution. ROS activate redox-sensitive pathways (MAPK, PI3K/AKT/mTOR, NF-κB, HIF-1α, Nrf2), regulating genomic stability, epigenetics, metabolism, inflammation, and the tumor microenvironment. Depending on ROS magnitude, duration, and context, outcomes are dual: tumor-promoting (proliferation, angiogenesis, metastasis, immune evasion, therapy resistance) or tumor-suppressive (apoptosis, senescence, oxidative catastrophe, anti-tumor immunity). Therapeutic strategies targeting ROS homeostasis include ROS scavengers, antioxidant modulators, mitochondrial-targeted antioxidants, redox-signaling inhibitors, pro-oxidant agents, and combination therapies that exploit oxidative stress vulnerabilities selectively in cancer cells.

mtROS signaling is also a valuable target, because an imbalance between ROS production and detoxification drives uncontrolled cancer cell proliferation and survival. At the same time, many cancer cells benefit from elevated mtROS through redox-dependent activation of oncogenic pathways, and they compensate for mitochondrial stress by upregulating antioxidant defenses and rewiring metabolism. These observations underscore that mitochondria, including their Ca^2^^+^ handling and MAM-associated signaling, play multifaceted roles in cancer metabolism and therapy response, with multiple potential targets but also substantial capacity for adaptation. Fig. 2 summarizes the ROS sources, signaling, biological outcomes, and therapeutic strategies in cancer.

**Table 2:** Mitochondria-targeted ROS-modulating agents in cancer.

Drug	Mechanism	ROS Modulation	Antitumor Evidence	Development Stage	Translational Limitations
IACS-010759	Potent complex I inhibitor; suppresses Oxidative Phosphorylation (OXPHOS) and increases electron leak at complex I [5].	↑ mtROS (superoxide → H_2_O_2_) at complex I.	*In vitro* and *in vivo* activity in OXPHOS-dependent models.	Early-phase clinical trials in selected malignancies.	Narrow therapeutic index; systemic toxicities; variable OXPHOS dependence.
MitoTam	Tamoxifen derivative conjugated to TPP; accumulates at complex I and blocks ubiquinone binding [121,167].	↑ mtROS at complex I; collapses ΔΨm.	Strong preclinical activity; phase I/Ib disease control in renal cell carcinoma.	Early clinical (phase I/Ib).	Limited clinical data; tissue-specific accumulation; need for larger trials.
MitoVES	TPP-conjugated vitamin E succinate; localizes to inner membrane; inhibits complex II [29].	↑ mtROS (superoxide) at complex II.	Robust pro-apoptotic effects in multiple tumor models.	Preclinical.	No clinical data; safety and delivery unresolved.
MitoQ	TPP-conjugated ubiquinone; mitochondrial antioxidant [163].	↓ mtROS (scavenges H_2_O_2_-linked oxidants).	Modulates bioenergetics and extracellular acidification; limited monotherapy antitumor effect.	Mostly preclinical in cancer; clinical use in non-oncology.	Potential to protect tumor cells; context-dependent effects.
MitoTEMPO	TPP-conjugated piperidine nitroxide; superoxide scavenger [162].	↓ mtROS (superoxide).	Alters tumor cell redox state; inconsistent suppression of growth/metastasis.	Preclinical.	Risk of blunting ROS-dependent therapy effects; requires careful positioning.
Mitochondria-targeted nanoplatforms	Nanoparticles functionalized with TPP/peptides to deliver ROS-generating or antioxidant payloads [130].	↑ or ↓ mtROS, depending on payload.	Proof-of-concept in cell and animal models.	Preclinical; no approved oncology use.	Complexity of manufacture, targeting, toxicity, and regulation.

## 8 Conclusions and Future Perspective

mtROS are central regulators of cancer initiation, progression, and therapeutic response, acting at the interface between oncogenic signaling, metabolic rewiring, and the tumor microenvironment. By integrating inputs from mtDNA alterations, nutrient and oxygen availability, and tumor–host interactions, mtROS can promote neoplastic transformation and metastatic spread or, when exceeding a critical threshold, trigger regulated cell death. Open questions regarding the identity of true mtDNA driver mutations, the impact of intratumoral heterogeneity in mitochondrial content and antioxidant capacity, and the effects of mtROS modulation on antitumor immunity, together with mixed clinical trial results, indicate that mtROS constitute a promising but context-dependent vulnerability. Future redox-stratified treatment strategies should incorporate quantitative assessment of tumor and host redox states, mitochondrial antioxidant capacity, and OXPHOS dependence to match pro-oxidant or antioxidant-leaning interventions to specific mtROS vulnerabilities while preserving normal tissue and immune function.

## Data Availability

Not applicable.

## Abbreviation

AKT	Protein kinase B
AMPK	Adenosine Monophosphate (AMP)-activated Protein Kinase
ATP	Adenosine Triphosphate
ATPIF1	ATPase inhibitory factor 1
BCL2	B-cell lymphoma 2
CSCs	Cancer Stem Cells
EMT	Epithelial-to-Mesenchymal Transition
ETC	Electron Transport Chain
FAD	Flavin Adenine Dinucleotide
FMN	Flavin Mononucleotide
GPx	Glutathione Peroxides
GRX	Glutaredoxin
GSH	Glutathione
IDH2	Isocitrate Dehydrogenase 2
IMS	Intermembrane Space
KEAP1	Kelch-like ECH-Associated Protein 1
MAMs	Mitochondria-Associated Membranes
MAPK	Mitogen-Activated Protein Kinase
MCU	Mitochondrial Calcium Uniporter
MICU1	Mitochondrial Calcium Uptake 1
miRNAs	microRNAs
MOMP	Mitochondrial Outer Membrane Permeabilization
MPT	Mitochondrial Permeability Transition
mtDNA	Mitochondrial DNA
mtROS	Mitochondrial ROS
NADH	Nicotinamide Adenine Dinucleotide
NADPH	Nicotinamide Adenine Dinucleotide Phosphate
NF-κB	Nuclear Factor Kappa-light-chain-enhancer of activated B cells
NOX	NADPH oxidases
NRF2	Nuclear factor erythroid 2-Related Factor 2
OXPHOS	Oxidative Phosphorylation
P2RX7	Purinergic Receptor P2X 7
P2RY2	Purinergic Receptor P2Y2
PET	Positron Emission Tomography
PI3K	Phosphatidylinositol 3-kinase
PML	Promyelocytic Leukemia
PTEN	Phosphatase and Tensin Homolog
RCD	Regulated Cell Death
ROS	Reactive Oxygen Species
SOD	Superoxide Dismutase
TCA	Tricarboxylic Acid
TRX	Thioredoxin
VEGF	Vascular Endothelial Growth Factor
